# The prevalence and determinants of active tuberculosis among diabetes patients in Cape Town, South Africa, a high HIV/TB burden setting

**DOI:** 10.1016/j.diabres.2018.01.018

**Published:** 2018-04

**Authors:** Natacha Berkowitz, Adaeze Okorie, Rene Goliath, Naomi Levitt, Robert J. Wilkinson, Tolu Oni

**Affiliations:** a Clinical Infectious Disease Research Initiative, Institute of Infectious Disease and Molecular Medicine, University of Cape Town, Cape Town 7925, South Africa; b Division of Public Health Medicine, School of Public Health and Family Medicine, Faculty of Health Sciences, University of Cape Town, Cape Town 7925, South Africa; c Division of Diabetes and Endocrinology, Department of Medicine, Groote Schuur Hospital, Cape Town 7925, South Africa; d The Francis Crick Institute Mill Hill Laboratory, London NW7 1AA, United Kingdom

**Keywords:** Tuberculosis, Risk factors, HIV/AIDS, Comorbidity, Screening, Sub–Saharan Africa, Diabetes, South Africa

## Abstract

**Aims:**

Studies addressing the association between diabetes mellitus (DM) and tuberculosis (TB) in sub–Saharan Africa are limited. We assessed the prevalence of active TB among DM patients at a primary care clinic, and identified risk factors for prevalent TB.

**Methods:**

A cross–sectional study was conducted in adult DM patients attending a clinic in Khayelitsha, Cape Town. Participants were screened for active TB (symptom screening and microbiological diagnosis) and HIV.

**Results:**

Among 440 DM patients screened, the active TB prevalence was 3.0% (95% CI 1.72–5.03). Of the 13 prevalent TB cases, 53.9% (n = 7; 95% CI 27.20–78.50) had no TB symptoms, and 61.5% (n = 8; 95% CI 33.30–83.70) were HIV–1 co–infected. There were no significant differences in either fasting plasma glucose or HbA_1c_ levels between TB and non–TB participants. On multivariate analysis, HIV–1 infection (OR 11.3, 95% CI 3.26–39.42) and hemoptysis (OR 31.4, 95% CI 3.62–273.35) were strongly associated with prevalent active TB, with no differences in this association by age or gender.

**Conclusions:**

The prevalence of active TB among DM patients was 4–fold higher than the national prevalence; suggesting the need for active TB screening, particularly if hemoptysis is reported. Our results highlight the importance of HIV screening in this older population group. The high prevalence of sub–clinical TB among those diagnosed with TB highlights the need for further research to determine how best to screen for active TB in high–risk TB/HIV population groups and settings.

## Highlights

• First in Africa to screen all DM patients for TB (irrespective of symptoms using Xpert) and HIV.
• Study found high prevalence of TB in DM patients, over half of whom were asymptomatic.
• HIV prevalence was 13% in DM patients, showing importance of HIV screening this older population.
• DM patients with HIV or hemoptysis at greater TB risk, and should be targeted for active TB case-finding.
• Given high subclinical active TB prevalence, screening should be irrespective of symptoms in this group.

## 1 Introduction

The Sustainable Development Goals (SDGs) [1] have made ending the global TB epidemic a priority and to achieve these new strategies need to be employed. Early detection of TB can lead to prompt treatment, increasing survival, and diminishing transmission risk. Interventions to identify TB in those at high risk for developing the disease are needed.

HIV–1 infection is the strongest recognized risk factor for TB, however other risk factors contribute to the TB burden, including smoking [2] alcohol abuse [3], vitamin D deficiency [4], and diabetes mellitus (DM) [5]. The growing DM epidemic has been associated with rapid urbanization and changing lifestyles in low and middle–income countries (LMIC), where the greatest increase in the number of people with DM and the greatest number of deaths due to DM are found. In a recent estimate, DM prevalence in sub–Saharan Africa (SSA) is projected to increase from 14.2 million cases in 2015 to more than double by 2040 [6]. This is emerging against the backdrop of a persisting high burden of infectious diseases including TB and HIV.

Previous studies have demonstrated that DM increases the risk of TB [7], [8], [9], [10], with DM associated with a 3–fold risk of TB compared to those without DM (95% CI 2.27–4.27) [5]. A systematic review on bi–directional screening of TB and DM cases concluded that active screening of DM patients leads to increased detection of TB cases [11]. TB prevalence among DM patients has been found to vary from 3% [12] to 36% [13]. While large–scale screening for TB amongst DM patients has been demonstrated to be feasible in routine and low–resourced healthcare settings [14], [15] the cost–effectiveness and validity of such strategies are unclear.

Studies investigating the prevalence of TB among persons with DM in the SSA context are limited [16], [17], [18]. In Tanzania, 1.3% of screened adults with DM had TB, 7–fold greater than the general population [16]. A modelling study estimated that up to 15% of TB cases in South Africa (SA) might be attributed to DM [19]. With TB incidence in SA remains one of the highest in the world with 834 cases per 100,000 people [20] and these studies demonstrate the potential to improve case detection through systematic screening of persons with DM, however the evidence to support this is lacking. A recent study of 672 patients with DM in Soweto, SA found a 4% prevalence of TB symptoms but no active TB cases amongst those with respiratory symptoms. Of note, only patients with clinical symptoms of TB were screened; and the influence of HIV infection was not explored [21]. Our study therefore aimed to investigate the prevalence of active TB, irrespective of the presence of symptoms, in this high HIV/TB co–infection setting.

## 2 Subjects, materials and methods

### 2.1 Study design and recruitment

Between September 2014 and October 2015, we conducted a cross–sectional study at a primary care clinic in Khayelitsha, a peri–urban township in SA. With a predominantly Black African population of 391,749 [22], it has one of the highest burdens of TB and HIV globally [23]. In 2012, the HIV antenatal prevalence in Khayelitsha was estimated at 34% (95% CI 31.0–36.6) (unpublished data from the 2012 Western Cape Department of Health Antenatal Survey) and the TB case notification rate was 1400 per 100,000 people annually, with 70% of TB cases being HIV–1 co–infected [24]. A community–based study in 2011 demonstrated a 13.1% (95% CI 11.0–15.1) prevalence of DM in Cape Town, specifically in the township communities [25].

To evaluate the prevalence of active TB in a routine clinical setting, consecutive adult DM patients attending a DM clinic for chronic disease management at a community health clinic were approached. Participants were recruited from a diabetes clinic, where their diagnosis had been previously made. As such, all patients attending this clinic had a pre–existing DM diagnosis, All adult patients (≥18 years at recruitment) attending this diabetes clinic were eligible to participate and their DM status was confirmed on review of patient records and treatment schedule. Patients were eligible to participate if they had a pre–existing DM diagnosis, were receiving DM treatment, and were aged ≥18 years at the time of recruitment. Participants were enrolled after written consent was given and then given a scheduled appointment to return, fasted, for sputum and blood collection.

The study received ethics approval from the University of Cape Town Human Research Ethics Committee (HREC Ref: 377/2015).

### 2.2 Data collection

Questionnaire: Research community health workers, bilingual in both English and Xhosa, the predominantly spoken languages in Khayelitsha, administered a questionnaire to collect socio–demographic and behavioral information using the WHO’s STEPwise approach to surveillance of chronic disease risk factors (STEPS) [26]. Participants were then scheduled to return within a week. A validated TB screening tool (adapted from the symptom–based Practical Approach to Lung Health and HIV/AIDS in SA [27]) was used to assess the presence of TB symptoms, including: cough, night sweats, fever, hemoptysis, and weight loss. In the same screening tool, information on TB contact history, previous history of TB, HIV status and self–reported chronic disease co–morbidity history were also collected. ART (Antiretroviral therapy) use was extracted from the clinic’s electronic database. Participants who did not know their HIV status were encouraged to undergo HIV testing, which consisted of a rapid point–of–care HIV antibody based test [28].

Measurements: At the return visit, following an overnight fast of 8–12 h, blood samples were collected for HbA_1c_ (glycated hemoglobin) and fasting plasma glucose (FPG) measurement. Participants were categorized as having “controlled” (HbA_1c_ <7% (53 mmol/mol)) and “poorly controlled,” (HbA_1c_ >7% [53 mmol/mol]) DM. Waist circumference, weight and height were measured and body mass index (BMI kg/m^2^) calculated as follows: underweight: <18.5; normal: 18.5 ≤ BMI < 25; overweight: 25 ≤ BMI < 30; obese: ≥30. Vital signs were also measured including respiratory and pulse rates, temperature, and blood pressure. Hypertension was defined as a systolic blood pressure (BP) ≥ 140 mmHg or diastolic BP ≥ 90 mmHg, a pre–existing diagnosis of hypertension, or taking medication for hypertension. TB screening and diagnoses were conducted using the national TB management guidelines [28]. Participants were classified as having subclinical TB if diagnosed with active TB but with an absence of any clinical symptoms. All participants underwent spontaneous or induced sputum collection. Sputum samples were processed according to national TB program guidelines [28]. This included GeneXpert processing and drug resistance testing for all participants, with additional smear microscopy if the GeneXpert result was positive or if participants were HIV–1–infected. An active TB case was defined as persons who tested positive for *M. tuberculosis* by either GeneXpert, smear microscopy, or TB culture in the presence or absence of clinical symptoms. An accredited national laboratory (National Health Laboratory Service) that adheres to standardized protocols and follows quality assurance measures processed collected samples. Patients diagnosed with active TB and/or HIV were referred for further clinical care and treatment.

### 2.3 Statistical analysis

The baseline characteristics were described using descriptive statistics and univariate analyses. Associations between participants with and without prevalent active TB were tested using Pearson’s χ^2^ test and Wilcoxon rank–sum test for categorical and continuous variables, respectively. The prevalence of active TB, including the confidence interval, was calculated.

Possible risk factors associated with active TB were further analyzed using multivariate logistic regression. Significance testing was two–sided at p–values ≤ 0.05. The multivariate model was built using purposeful selection where risk factors were selected based on clinical and statistical significance to control for possible confounders [29]. Odds ratios (OR) and 95% confidence intervals were also estimated. Model validation was performed to identify any outliers and influential observations and the fit of the model to the data was evaluated using Pearson’s goodness of fit test. Potential effect modification was assessed using interaction variables. A sensitivity analysis was conducted to investigate the potential impact of unknown HIV status. All data analyses were conducted using STATA version 13 (StataCorp, College Station, TX, USA).

## 3 Results

**Fig. 1:**
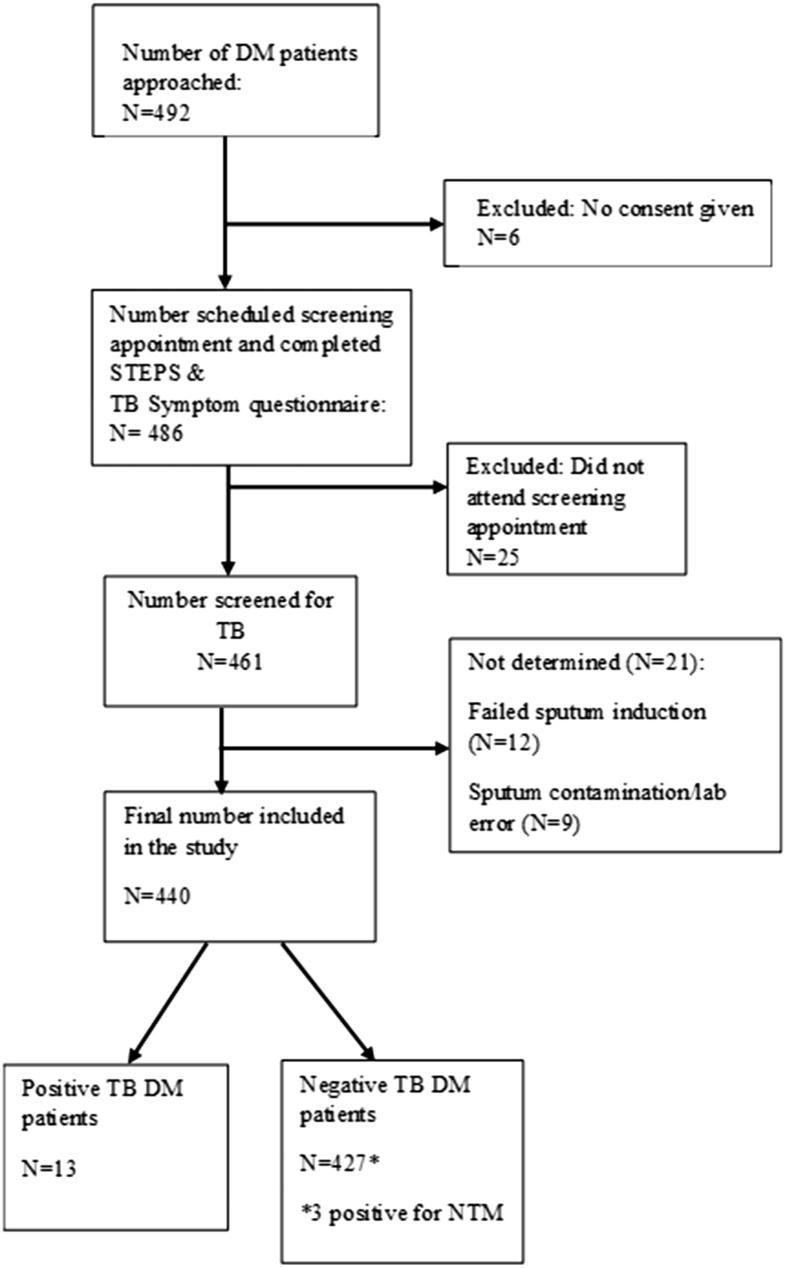
Flowchart of recruitment process and results of screening DM patients for TB attending a DM clinic at Khayelitsha (Site B) Community Health Clinic from September 2014 to October 2015. DM = diabetes mellitus; TB = tuberculosis.

We approached 492 DM patients to participate in the study, of whom 52 were excluded, resulting in a final sample size of 440 (Fig. 1). Reasons for exclusion include: consent refusal (n = 6), missed attendance of follow–up appointment (n = 25), and indeterminate/contaminated sputum results (n = 21). Demographic (Table 1) and baseline clinical (Table 2) characteristics were stratified by TB status. The median age of the included sample was 54.6 years and 75% were female, with no statistically significant difference in age (55.2 years; IQR 45.52–60.21 years versus 54.6 years; IQR 46.86–62.12 years) and proportion female (61.5%; 95% CI 33.27–83.70 versus 38.5%; 95% CI 16.30–66.73) between TB cases and non–cases respectively.

**Table 1:** Demographic characteristics of 440 DM patients with and without TB attending a DM clinic.

Characteristic¶	TB^a^	Non–TB	Total Screened^b^	P-value^c^
# Of subjects	13	427	440	
Age (y)^d^	55.2 [45.52–60.21]	54.7 [46.86–62.12]	54.6 [46.86–62.02]	0.766
Gender (females)	8 (61.5)	322 (75.4)	330 (75.0)	0.277
HIV status				<0.001*
Positive	8 (61.5)	48 (11.2)	56 (12.7)	
Negative	5 (38.5)	292 (68.4)	297 (67.5)	
Unknown	0 (0.0)	87 (20.4)	87 (19.8)	
Employment status (n = 437)				0.650
Employed	5 (38.5)	138 (32.6)	143 (32.7)	
Unemployed	6 (46.2)	175 (41.3)	181 (41.4)	
Retired	2 (15.4)	111 (26.2)	113 (25.9)	
Smoking^e^ (n = 439)	2 (15.4)	76 (17.8)	78 (17.8)	0.816
Alcohol use^e^, n = 439)	3 (23.1)	63 (14.8)	66 (15.0)	0.600
Average monthly income (ZAR) (n = 381)	1750 [1300–3350]	1400 [1300–2600]	1400 [1300–2600]	0.042

¶ Unless otherwise noted data presented as n, n (%).

* p < .05.

a TB: tuberculosis.

b Sample size indicated where data missing.

c Logistic regression analysis.

d Data presented in median (interquartile range).

e Past or current.

**Table 2:** Baseline clinical characteristics of 440 DM patients with and without TB attending a DM clinic.

Clinical characteristic¶	TB^a^	Non–TB	Total screened^b^	P-value^c^
# of subjects	13	427	440	
TB contact	2 (15.4)	106 (24.8)	108 (24.6)	0.413
Gender (females)	8 (61.5)	322 (75.4)	330 (75.0)	0.277
Previous TB	2 (15.4)	84 (19.7)	86 (19.6)	0.693
Positive smear	2 (15.4)	0 (0.0)	2 (0.5)	
Positive culture	1 (7.7)	0 (0.0)	1 (0.2)	
Any TB symptom	6 (46.2)	199 (46.6)	205 (46.6)	0.974
1–2 symptoms	3 (23.1)	138 (14.5)	141 (32.1)	
≥3 symptoms	3 (23.1)	61 (32.1)	64 (14.6)	
Cough > 2 weeks	3 (23.1)	55 (12.9)	58 (13.2)	0.324
Fever	3 (23.1)	37 (8.7)	40 (9.1)	0.126
Weight loss	3 (23.1)	71 (16.6)	74 (16.8)	0.557
Fatigue	2 (15.4)	135 (31.6)	137 (31.1)	0.185
Blood–stained sputum	2 (15.4)	4 (0.9)	6 (1.4)	0.008
Chest pain	2 (15.4)	45 (10.5)	47 (10.7)	0.598
Night sweats	2 (15.4)	70 (16.4)	72 (16.4)	0.922
Hypertension (n = 435)	7 (53.8)	255 (60.4)	262 (60.2)	0.636
Triglycerides (mg/dL) (n = 436)	1.4 [1.3–2.0]	1.3 [1.0–1.7]	1.3 [1.0–1.8]	0.108
BMI^a^^,^^d^ (kg/m^2^) (n = 434)	28.6 [25.11–45.08]	32.8 [28.60–38.64]	32.7 [28.37–38.73]	0.895
≤18.5 (underweight)	0 (0.0)	3 (0.7)	3 (0.7)	0.314
18.5–24.9 (normal)	3 (23.1)	48 (11.4)	51 (11.8)	
25–29.9 (overweight)	4 (30.8)	94 (22.3)	98 (22.6)	
≥30 (obese)	6 (46.2)	276 (65.6)	282 (65.0)	
Diabetes control (n = 435)				
Poorly controlled	12 (92.3)	336 (79.6)	348 (80.0)	0.212
HbA_1c_^a^^,^^d^^,^^e^ (n = 435)	9.5 [8.20–9.90]	9.1 [7.30–11.00]	9.2 [7.30–11.00]	0.897
4.8–7.7% (6.7 ± 0.69)	2 (15.4)	143 (33.9)	145 (33.3)	0.212
7.8–10.3% (9.1 ± 0.80)	9 (69.2)	138 (32.7)	147 (33.8)	
10.4–18.4% (12.0 ± 1.33)	2 (15.4)	141 (33.4)	143 (32.9)	
Fasting plasma glucose^d^^,^^e^ (mmol/L) (n = 434)	8.2 [6.3–10.4]	8.2 [6.1–11.7]	8.2 [6.1–11.6]	0.807
2.7–6.8 (5.4 ± 0.97)	5 (38.5)	145 (34.4)	150 (34.6)	0.821
6.9–10.4 (8.5 ± 1.06)	5 (38.5)	136 (32.3)	141 (32.5)	
10.5–23.5 (13.8 ± 2.66)	3 (23.1)	140 (33.3)	143 (33.0)	

¶ Unless otherwise noted data presented as n, n (%).

a BMI: body mass index; HbA_1c_: glycated hemoglobin; TB: tuberculosis.

b Sample size indicated where data is missing.

c Logistic regression analysis.

d Data presented in median (interquartile range).

e Categories presented in tertiles (mean ± standard deviation).

### 3.1 Prevalence of active TB

Active TB prevalence was 3.0% (95% CI 1.72–5.03). Among non–TB cases, 3 were positive for non–TB mycobacteria (NTM) but given the lack of symptoms were classified as non–TB cases. The majority of active TB cases (84.6%; 95% CI 53.30–96.36) were detected by GeneXpert; with 15.4% (95% CI 3.64–46.70) of these being smear microscopy positive. Only one case (7.7%; 95% CI 0.98–41.20) was diagnosed through *M.tb.* culture alone, testing negative on both GeneXpert and smear microscopy tests. The prevalence of HIV was significantly higher in TB cases (61.5%; 95% CI 33.27–83.70) compared to non–TB cases (11.2%; 95% CI 8.57–14.62). 19.8% (n = 87) of participants did not have HIV status ascertained and declined testing.

### 3.2 TB symptoms

Among study participants, 46.6% (95% CI 41.98–51.29) reported at least one TB symptom; with no significant difference in the prevalence of any TB symptoms between TB (46.1%; 95% CI 21.55–72.79) and non–TB cases (46.6%; 95% CI 41.90–51.37). Among all participants, 14.6% (n = 64) reported three or more symptoms. Among TB cases, 23.1% (n = 3) reported three or more symptoms. Cough, fever, and weight loss were the most commonly reported symptoms for active TB cases and were equally reported (each symptom: 23.1%; 95% CI 7.24–53.56). Fatigue (31.6%; 95% CI 27.36–36.20), weight loss (16.6%; 95% CI 13.38–20.48), and night sweats (16.4%; 95% CI 13.16–20.23) were the most common symptoms reported among non–TB cases. Of note, among TB cases, 53.9% (95% CI 27.21–78.45) were completely asymptomatic for TB.

Of the subclinical TB cases (all GeneXpert positive) (n = 7), 57.1% of the participants were smear negative (n = 4), and one had a confirmed positive culture (14.3%). Two participants reported having TB previously (28.6%). None reported having any previous TB contact or exposure. One participant reported a history of smoking (14.3%). Most of the subclinical cases were female (n = 4, 57.1%) and the median age of subclinical patients was 55.2 years [IQR 45.52–60.21 years]. The majority of subclinical cases (71.4%) were HIV positive (n = 5).

### 3.3 DM management

The overall median HbA_1c_ was 9.3% (78.0 mmol/mol) (IQR 7.30–11.00%) (IQR 56.00–97.00 mmol/mol) and fasting plasma glucose (FPG) was 9.2 mmol/L (IQR 6.10–11.60 mmol/L). In Table 2, HbA1_c_ and FPG levels were divided into tertiles as follows: lower, middle, and upper tertiles. Among active TB cases, 69.2% had HbA1_c_ levels in the middle tertile (7.8–10.3%). There was no significant difference in either FPG (p = .821) or HbA_1c_ (p = .212) levels between TB and non–TB cases. The majority of DM patients (80.0%; 95% CI 75.96–83.51) had poorly controlled DM despite being on DM medication. Although not statistically significant (p = .314), a greater proportion of non–TB cases (65.6%; 95% CI 60.87–69.96) were classified as obese compared to TB cases (46.2%; 95% CI 21.55–72.79). Hypertension was the most common co–morbidity among all participants (60.2%; 95% CI 55.53–64.74), compared to other self–reported co–morbidities such as asthma/COPD (4.1%; 95% CI 2.59–6.41) and depression (0.1%; 95% CI 0.02–2.10).

### 3.4 Risk factors associated with prevalent active TB

On univariate analysis, HIV (X^2^ 21.1, p < .001), ART medication (X^2^ 21.5; p < .001), average monthly income (p < .001) and hemoptysis (X^2^ 19.6; p < .001) were associated with prevalent active TB. To facilitate comparison of nested models, multivariate analyses were performed excluding participants with unknown HIV status. In the adjusted model (Table 3), the odds of having active TB were 11.3 times higher for HIV–1 infected DM patients (95% CI 3.26–39.42), and patients reporting hemoptysis were 31 times more likely to have active TB (adjusted OR: 31.4; 95% CI 3.62–273.35).

**Table 3:** Univariate and multivariate analyses of risk factors associated with TB.

Characteristic	OR^a^ (95% CI)	P–value	AOR^b^ (95% CI)	P–value
*HIV (n = 353)*				
Yes	9.7 (3.06–31.00)	<0.001	11.3 (3.26–39.42)	<0.001
No	1.0		1.0	

ART medication				
Yes	9.6 (3.06–30.14)	<0.001		
No	1.0			

Blood–stained sputum				<0.001
Yes	19.2 (3.18– 116.30)	0.008	31.4 (3.62– 273.35)	
No	1.00		1.00	

*Fatigue*				
Yes	0.4 (0.09–1.80)	0.185		
No	1.0			

*Fever*				
Yes	3.2 (0.83–11.10)	0.126		
No	1.0			

*DM control*				
Poorly controlled DM	3.1 (0.39–24.00)	0.212		
Controlled DM	1.0			

*Gender*				
Female	0.5 (0.17–1.63)	0.277		
Male	1.0			

*Family history of DM*				
Yes	2.0 (0.60–6.47)	0.253		
No	1.0			

*Average monthly income (n = 381)*	1.0	0.042		

*Triglycerides (mg/dL) (n = 436)*	1.4 (0.98–1.97)	0.108		

*BMI (kg/m^2^) (n = 434)*				
<18.5	1.0	0.314		
18.5–24.9	1.5 (0.32–6.83)			
25–29.9	1.0			
>30	0.5 (0.14–1.85)			

*HbA_1c_ (%) (n = 435)*				
<7	1.0	0.212		
>7	3.07 (0.37–22.60)			

a OR: odds ratio.

b AOR: adjusted odds.

The odds of having HIV among DM patients with TB were nearly 10 times higher (OR 9.7; 95% CI 3.05–31.00) compared to DM patients without TB. There were no statistically significant interactions with age, gender, smoking status, hypertension, and DM control variables.

### 3.5 Sensitivity analysis of HIV status

HIV status was self–reported at enrolment. Among all participants, 19.8% (n = 87) did not know their HIV status. Among those that reported a negative or unknown status (n = 392), 77.8% (n = 305) agreed to undergo HIV testing. Among those tested, eight participants were newly diagnosed with HIV. We conducted sensitivity analysis (Table 4) to investigate the potential impact of excluding participants with unknown HIV status. Re–classification of HIV status unknown as HIV negative did not change the significant variables in the multivariate model, but increased the adjusted OR (aOR) for HIV to 14.8 (95% CI 4.25–51.17), and marginally decreased the aOR for hemoptysis to 30.3 (95% CI 3.72–247.43). Re–classifying HIV unknown patients as positive resulted in a significant aOR decrease of 3.7 (95% CI 1.14–11.92) for HIV and an aOR of 21.8 (95% CI 3.29–144.86) for hemoptysis.

**Table 4:** Sensitivity analysis of “unknown” HIV status.

HIV risk factor	OR^a^ (95% CI)	AOR^b^ (95% CI)	P–value
“Unknown” HIV status reclassified as “negative” (n = 440)	12.63 (3.97–40.18)	14.75 (4.25–51.17)	<0.001

Excluded “Unknown” HIV status (n = 353)	9.73 (3.06–31.00)	11.34 (3.26–39.42)	<0.001

a OR: odds ratio.

b AOR: adjusted odds.

## 4 Discussion

The main findings of this study were that the prevalence of active TB among DM patients at 3.0% was 4.3–fold greater than the national estimate of 696 per 100,000 [20]. DM patients with HIV–1 infection were 11 times more likely to have active TB than those without HIV; hemoptysis was significantly associated with prevalent active TB and there was a lack of association between glycemic status and active TB.

The prevalence rate of active TB in our study was within the range of 0.1–6.2%; reported prevalence rates from previous screening studies conducted in similar high TB burden settings such as Tanzania and Ethiopia [16], [17]. However, our study differs from many others in that all DM patients were screened for active TB irrespective of reported symptoms. In similar screening studies, sputum samples were collected from only DM patients clinically suspected of having TB [15], [16], [30]. Previous evidence has shown that symptom screening may not be an effective method in detecting TB in those with HIV [31], [32] and DM [33]. Our findings support this, as 54% of TB cases had no TB symptoms. This was similar to a study conducted in a comparable setting where a high prevalence of sub–clinical TB was found among HIV patients [30], with more than half of these cases developing symptoms 3 days to 2 months later. This highlights the importance of early active TB case detection in sub–clinical patients, especially those co–infected with HIV and those with co–morbid with DM. Additionally, we noted that classic TB symptoms were not significantly different between active TB and non–TB cases. This alludes to the poor specificity of these symptoms in persons with DM, with symptoms such as fatigue and weight loss also representing symptoms of DM.

Other studies have reported the risk of active TB to be higher in DM males compared to females [30], [34], [35], [36], [37]. In our study, whilst the odds of having active TB was greater in females than males, this difference did not achieve statistical significance.

In our study, elevated HbA_1c_ and FPG (using cut–off values of 7% [53 mmol/mol] and 7 mmol/L respectively) were not statistically significant risk factors for prevalent active TB. This was comparable to other studies evaluating HbA_1c_ that found no association [38], [39]. However, other studies have reported an association between TB and HbA1_c_ levels [30], [40], particularly in patients with HbA1_c_ >9.0% (75 mmol/mol) [30]. In one cohort study, there was a linear relationship between FPG and TB risk [41]. These differences may be due to the different measures (HbA_1c_, FPG) and cut–off values used to define glycemic control. For example, using quintiles to explore the association between HbA_1c_ and TB may reveal a higher prevalence of active TB at much higher HbA_1c_ levels that may be masked using a binary categorization. However, due to the small sample of TB cases, our study was underpowered to explore this. In addition, FPG may be more likely to reflect stress–induced hyperglycemia than HbA_1c_. Given these inconsistencies between studies, proxies of optimal DM management have been explored as an alternative method to further evaluate the association between poor glycemic control and TB risk. In one longitudinal study conducted in a low TB burden setting, whilst there was no association found, those with the highest and lowest numbers of consultations or clinic follow–ups had the strongest association with TB risk, compared to other significant risk factors [38]. This finding suggests that measures of DM control that take into consideration poor or excessive attendance at the DM clinic could be valuable measures to better assess DM control and subsequent TB risk.

While the prevalence of HIV among DM patients screened for this study (12.7%) was similar to the national SA prevalence rate at 12.6% [42], the prevalence of patients with both DM and TB was nearly 5 times greater than the national and study population rate. DM participants with TB were more likely to have HIV compared to DM participants without TB as the odds were nearly 10 times higher compared to patients without TB.

It was surprising that smoking and alcohol dependence, well–known risk factors for TB [43], [44], were not identified as such in our study. As smoking rates are higher in males, the higher proportion of female participants in this study is likely to have reduced the statistical power of our study to detect this association. Overall our study revealed a high prevalence of smoking (18%), which was similar to the SA national prevalence 17.6% [45], emphasizing the need to address modifiable risk factors as part of an integrated preventative approach to improving chronic disease outcomes. Several limitations were identified in this study. Selection bias may have been present as inclusion required participants to return for a scheduled appointment. However, as only 25 participants did not return, this is unlikely to significantly influence the results. Additionally, our participants were recruited from a DM clinic and thus our results may underestimate the prevalence of TB in undiagnosed or non–adherent DM patients. Since the study was underpowered, we were unable to explore the effect of additional factors such as glycemic control, duration of DM diagnosis and medication use on TB risk; consequently, these variables were not collected.

Reported symptoms were not confirmed or validated. There is a possibility that patients might have misinterpreted or misperceived definitions of TB symptoms that might have resulted in an over– or under–estimation of reported symptoms leading to respondent/recall bias. Apart from cough and fever, the temporality and duration of these classical TB symptoms were also not reported. For TB contact, date of contact was not obtained and so it was not possible to ascertain the importance of duration since contact on risk of active TB. Potential confounders such as vitamin D and consultation rates, were not measured. As such there is potential for error due to these unmeasured confounders. Lastly the wide confidence intervals of HIV and hemoptysis in the multivariate analysis produce uncertainty and a lack of precision about the true magnitude of association with active TB.

Nonetheless, the prevalence of active TB in DM patients was much higher than the national estimate in the general population, demonstrating that screening approaches targeting DM patients are potentially more efficient than screening the general population. This is particularly so in DM patients with HIV–1 infection who are at an even greater risk of TB.

The importance of HIV–1 as a significant risk factor for TB has been further confirmed in DM patients in this study. This finding also shows the growing trend of multi–morbidity, and the need to focus on the care and management of both chronic infectious and non–communicable diseases, including early diagnosis and treatment of HIV in (often older) patients with diabetes.

Whilst the presence of hemoptysis was associated with a higher risk of prevalent active TB, the majority of active TB cases were asymptomatic at the time of diagnosis. This finding poses a serious threat to TB control as conventional symptom screening could delay TB diagnosis in this target population, as a significant proportion of DM patients with TB would be missed on routine screening. The low sensitivity of symptom screening highlights the need for accurate point–of–care diagnostic tools such as the GeneXpert to detect asymptomatic TB cases.

For this target population, further studies are required to investigate who should be screened to improve screening feasibility needs to be considered. Should we consider routine screening for TB amongst all DM patients in high TB prevalence settings? Or should screening focus on DM–HIV patients only? A significant decrease in TB incidence and mortality is required if the SDG and WHO goals to eliminate TB are to be met. Further research is therefore required to determine the most cost–effective and accurate TB screening algorithms to increase early case detection, as well as evaluating the impact of implementing TB screening on TB outcomes in this high–risk population.
